# Effect of natural products on host cell autophagy induced by Influenza A virus infection

**DOI:** 10.3389/fcimb.2024.1460604

**Published:** 2024-09-30

**Authors:** Xiaopan Liu, Qingsen Wang

**Affiliations:** ^1^ Institute of Animal Husbandry and Veterinary Medicine, Fujian Academy of Agricultural Sciences/Fujian Key Laboratory of Animal Genetics and Breeding, Fuzhou, China; ^2^ Ministry of Education Key Laboratory for Avian Preventive Medicine, Yangzhou University, Yangzhou, China

**Keywords:** Influenza A virus, autophagy, plant extracts, viral infections, antiviral therapies

## Abstract

Influenza A virus (IAV) can cause seasonal epidemics and global pandemics, posing serious threats to public health, making a deeper understanding of its biological characteristics and effective countermeasure strategies essential. Autophagy not only maintains cellular homeostasis but also plays an important role in host defense against IAV infection. However, the relationship between IAV and autophagy is complex, and effective antiviral drugs are not yet available. Natural products have shown excellent potential in disease control due to their diversity and multi-targeting. This review focuses on the relationship between IAV and autophagy and discusses the potential of targeting autophagic pathways for the development of new antiviral therapies. Particularly, the use of plant extracts as autophagy modulators has garnered attention due to their non-toxic nature and cost-effectiveness, which provides strong support for the development of future antiviral drugs that can help to inhibit viral infections and slow down disease progression.

## 1 Introduction

Autophagy is a cellular process of self-degradation and recycling (Glick et al., 2010). When cells are stimulated by various stress factors, they can generate specialized vesicles that encapsulate cellular components such as damaged organelles, lipid droplets, superfluous proteins, and pathogenic microorganisms (He and Klionsky, 2009). These vesicles elongate, curve, and eventually close to form autophagosomes, which then fuse with lysosomes for breakdown and degradation. This degradation process helps maintain the stability of the intracellular environment. In mammalian cells, autophagy is broadly categorized into three main types: macroautophagy, microautophagy, and molecular chaperone-mediated autophagy (Levine et al., 2011). Among these, macroautophagy is the predominant form (Kaur and Debnath, 2015; Levine et al., 2011). Recent research has further classified autophagy into selective and non-selective types based on its specificity for degrading substrates (Yamamoto and Noda, 2020). Non-selective autophagy involves the random transport of cytoplasmic components to lysosomes, whereas selective autophagy targets specific substrate proteins for degradation (Chung et al., 2020; Li et al., 2019). Depending on cellular conditions and the presence of stimuli, autophagy can also be classified as induced autophagy and basal constitutive autophagy (Inuzuka et al., 2009). Basal autophagy operates at a low, continuous level in most cells, crucial for the routine turnover of intracellular substances and maintenance of cellular homeostasis. In contrast, induced autophagy occurs at a heightened level in response to external stressors, serving as a protective mechanism to rapidly elevate cellular degradation processes (Sun et al., 2018).

**Figure 1:**
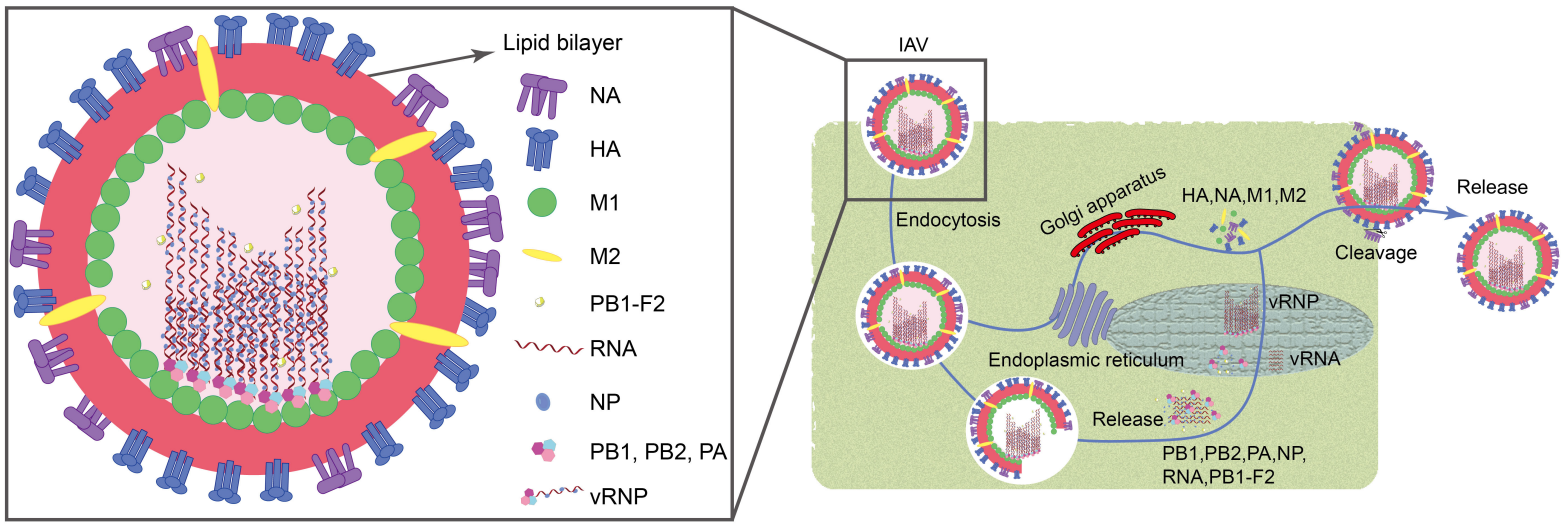
Structure and cellular invasion mechanism of IAV.

Influenza A virus (IAV) is associated with high morbidity and mortality. They are extremely pathogenic and cause significant economic losses, posing a constant threat to public health (Ampomah and Lim, 2020). IAV belongs to the family Orthomyxoviridae and typically exhibits filamentous or spherical morphology with a diameter ranging from 80 to 120 nm (Horiguchi et al., 2019). These viruses are characterized as single-stranded, negative-sense RNA viruses with a genome length of approximately 13.6 kb. They encode a total of 11 viral proteins, including essential polymerase proteins (PB1, PB2, PA, and PB1-F2), hemagglutinin (HA), neuraminidase (NA), nucleoprotein (NP), matrix proteins (M1 and M2), and non-structural proteins (NS1 and NS2) (Luo, 2012; Li et al., 2024). When the virus invades, its surface HA binds to sialic acid residues on the host cell surface, forming a swallowing vesicle, and enters the host cell via endocytosis (Jin et al., 2017). Subsequently, the M2 ion channel opens, causing the viral envelope to fuse with the engulfing vesicle, releasing the nucleocapsid into the cytoplasm through the M1 channel. Under the action of NP, PA, PB1, PB1-F2, and PB2, the nucleocapsid enters the host cell nucleus. Inside the nucleus, newly synthesized negative-sense RNA (vRNA) binds to NP, PB1, PB2, and PA to form the ribonucleoprotein complex (vRNP). Simultaneously, HA, NA, M1, and M2 synthesized in the host cell’s endoplasmic reticulum and Golgi apparatus are utilized for assembly at the cell membrane with vRNP (Zou et al., 2022; Li et al., 2024). Finally, viruses are cleaved from the cell membrane by the enzymatic activity of the NA protein, completing the release process (**Figure 1**).

When host cells are attacked by viruses, the autophagy mechanism is activated as a defense strategy that helps to recognize, encapsulate and degrade viral particles, thereby limiting viral replication and spread (Ammanathan et al., 2020; Dreux and Chisari, 2009). Viral infection can promote the increase of autophagy in host cells through multiple pathways, including direct manipulation of the expression of autophagy-related genes, regulation of autophagosome formation and degradation processes, as well as indirectly by interfering with cellular metabolism, activating immune responses, or triggering cellular stress (Roldán et al., 2019; Zhang et al., 2023). For example, the IAV, measles virus and human immunodeficiency virus type I are capable of inducing autophagy (Hoenigsperger et al., 2024; Wang et al., 2012). However, herpes simplex virus type I and human cytomegalovirus can inhibit autophagy (Ripa et al., 2024; Li et al., 2024). The complex interactions between viruses and autophagy are receiving extensive attention from the scientific community, and targeting autophagy is expected to be a new strategy for antiviral therapy.

Natural products have long been widely used in the development of antiviral drugs because of their multi-targeting and low side effects (Xiao et al., 2018; Xu et al., 2020). Research reveals the complex relationship between these compounds and autophagy. For instance, andrographolide has been shown to promote autophagy by the RAGE/PI3K/AKT/mTOR pathway, thereby ameliorating sepsis-induced acute lung injury (Qin et al., 2024), while saikosaponin D has been found to inhibit the proliferation and metastasis of colorectal cancer cells by increasing LC3B and p62 autophagic factor levels (Lee et al., 2024). In addition, more and more studies have shown that natural products can exert antiviral effects by regulating autophagy (Liu et al., 2018; Xing et al., 2021; Vidoni et al., 2022). Statistics show that from 1981 to 2019, 186 antiviral drugs have been successfully approved, including 49 antiviral drugs related to natural products (Newman and Cragg, 2020). However, compared to the huge number of biological species in nature, there are still a large number of natural products with potential antiviral abilities that have not yet been discovered. Therefore, exploring natural products remains an important direction for current antiviral drug development. With the advancement of chemical and pharmacological technologies, the application of these natural compounds has gradually become ordered and standardized, providing new perspectives for drug discovery (Liu et al., 2022). In this review, we will summarize the relationship between autophagy and viruses and explore the potential pharmacological mechanisms by which natural products exert antiviral effects through modulating autophagy, to provide a reference for the development of novel antiviral drugs.

## 2 Autophagic processes and detection

### 2.1 Autophagy

**Figure 2:**
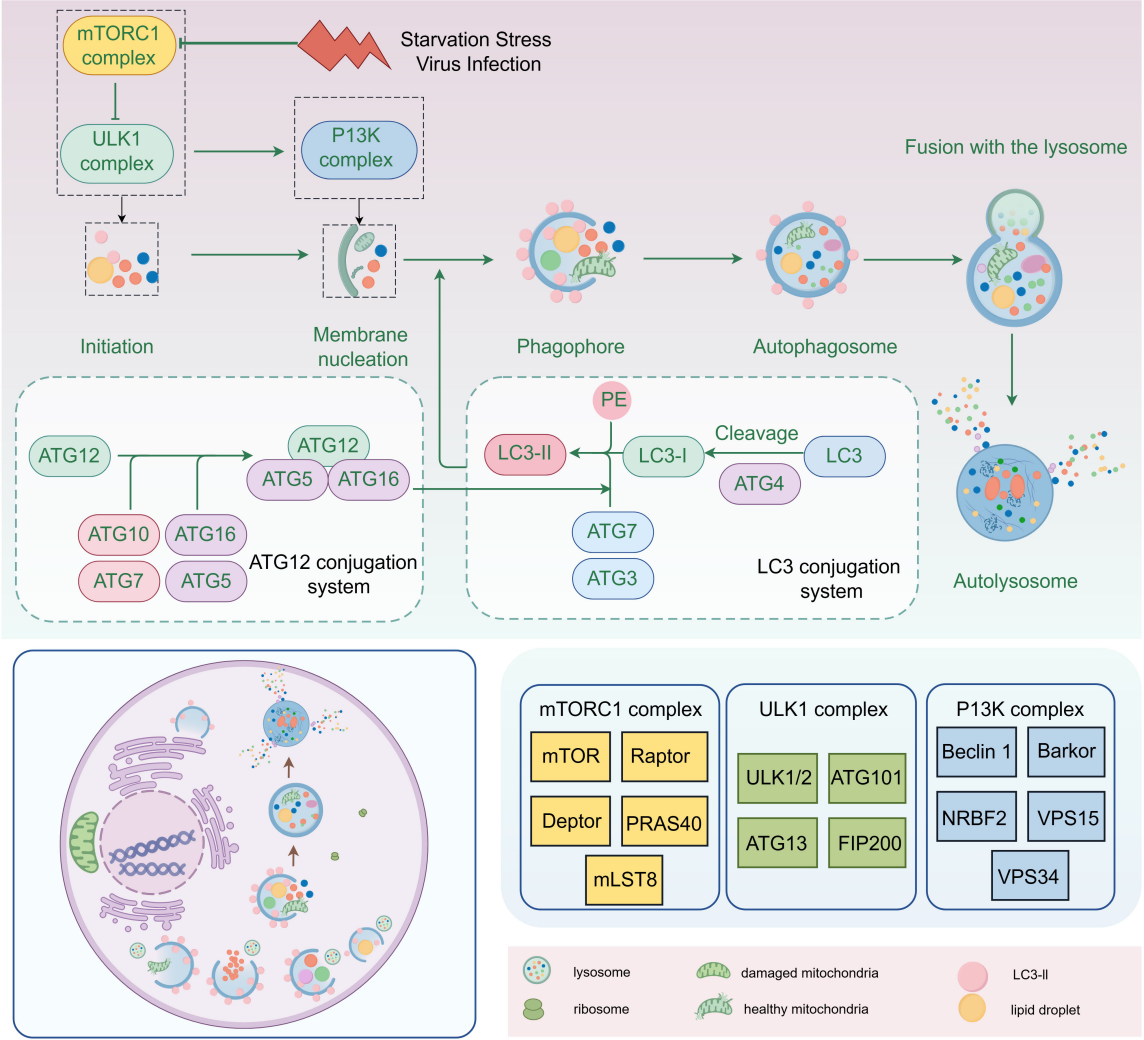
The process of intracellular autophagy and its related proteins (By Figdraw).

The essence of autophagy is the process of rearrangement of intracellular membranes, a process that includes initiation, vesicle formation, vesicle extension and closure, fusion with lysosomes, and disassembly (Kaur and Debnath, 2015). During the initiation phase, the mTORC1 complex (composed of mTOR, Raptor, Deptor, PRAS40 and mLST8) regulates the activation of autophagy. During vesicle formation, the ULK complex (composed of ULK1/2, Atg101, Atg13 and FIP200) and PI3K complex (composed of Beclin 1, Barkor, NRBF2, VPS15 and VPS34) play key roles. Vesicle extension and closure are dependent on the Atg12-Atg5-Atg16 system and the LC3 system, with the lipidation of LC3-II being the hallmark event of this process (**Figure 2**). Mature autophagosomes fuse with lysosomes to form autolysosomes, a fusion process that involves molecules such as SNARE proteins. Finally, in autophagic lysosomes, the encapsulated material is broken down by lysosomal enzymes, releasing essential molecules that are reused by the cell (Chen et al., 2023; Dossou and Basu, 2019; Xi et al., 2019).

### 2.2 Detection of autophagy

There are numerous methods for the detection of autophagy, and each technique has its unique advantages and limitations (Mizushima et al., 2010). Western blotting (WB) is effective for assessing autophagic activity by measuring the expression levels of autophagy-related proteins such as LC3-II/I and p62 (Mizushima and Murphy, 2020). Mang used WB analysis to show that the M2 protein in IAV inhibits the autophagy process by assessing the expression levels of p-Akt, Akt, p-mTOR, mTOR, becn1, ATG5, and LC3-I/LC3-II proteins (Wang et al., 2019a). Immunofluorescence uses fluorescently labeled autophagy proteins to observe autophagic vesicle formation, and the researchers used LC3 labeled with green fluorescent protein (GFP-LC3) to observe autophagic vesicle formation by fluorescence microscopy (Tang et al., 2012). Moreover, the unique properties of Keima proteins, which exhibit spectral changes under neutral and acidic conditions, make them a powerful tool for monitoring the process of autophagy from cytoplasm to lysosomes. This assay applies only to living cells and can be analyzed using fluorescence zymography or fluorescence microscopy (Nagata et al., 2018). According to reports, Zhang et al. utilized mito-Keima for immunofluorescence to assess mitochondrial autophagy levels, demonstrating that the NP protein encoded by the PR8 virus induces mitochondrial autophagy (Zhang et al., 2023).

In addition, the researchers developed a GFP-RFP-LC3 dual-color fluorescent fusion protein system that exploits the pH differences between autophagosomes and autolysosomes. In autophagosomes, where GFP and RFP coexist, fluorescence microscopy shows yellow fluorescence. In the acidic environment of autolysosome, GFP fluorescence is quenched, leaving only red fluorescence from RFP (Chen et al., 2023; Sun et al., 2018). Although fluorescence microscopy can visualize and localize autophagy, its quantification remains challenging. Transmission electron microscopy (TEM), with its high resolution, can observe the autophagy process in detail and provide direct visual evidence but is technically demanding and expensive (Liu et al., 2017). Beale et al. utilized TEM and immunofluorescence to discover that M2 disrupts autophagy by binding to the highly conserved LIR motif (LC3-interacting region) in the cytoplasm, thereby promoting filamentous budding and viral particle stability (Beale et al., 2014). Flow cytometry can detect autophagic activity using the ratio of GFP to RFP. The GFP-RFP-LC3 fusion protein expressed in cells is processed by Atg4 endopeptidase and breaks into GFP-LC3 and RFP-LC3ΔG, where the shift in the ratio of GFP-LC3 as an autophagic substrate to the internal control RFP/LC3ΔG reflects the level of autophagic activity (Date et al., 2022). Among these methods, WB, TEM, and immunofluorescence are commonly used. In recent years, several studies have also begun to use flow cytometry to detect autophagy levels, but it is still relatively rarely used compared to other methods.

In recent years, emerging technologies such as proteomics, metabolomics, transcriptomics, and network pharmacology have significantly advanced autophagy research. Metabolomics provides crucial insights into metabolic changes during autophagy by analyzing key metabolites, including amino acids, carbohydrates, and lipids (Stryeck et al., 2017). Transcriptomics elucidates the expression patterns of autophagy-related genes and their regulatory mechanisms (Jiao et al., 2020), while proteomics offers detailed information on protein alterations associated with autophagy by identifying and quantifying relevant proteins (Cudjoe et al., 2017). The integration of these technologies has unveiled key molecules and mechanisms involved in autophagy. For instance, the combined analysis of metabolomics and proteomics identified phospholipase A2 as a critical target in autophagy related to gouty arthritis (Fu et al., 2023). Additionally, transcriptomics has highlighted the role of autophagy in the infection process of the pepper mild mottle virus (Jiao et al., 2020). Network pharmacology has also been instrumental in predicting drug targets related to autophagy, such as HSPA5 and PARP1 (Guo et al., 2024), which are crucial for regulating lysosomes and autophagosomes and are involved in viral replication. Overall, the synergistic application of these techniques not only enhances the understanding of autophagy mechanisms but also advances research in related diseases and drug development.

## 3 IAV infection and cellular autophagy

The relationship between autophagy and IAV is complex. The specific mechanism of influence between different pathogens and autophagy can be based on the genome sequence, antigenicity, pathogenicity, replication efficiency, and transmissibility of specific pathogens (Khandia et al., 2019). Autophagy was closely associated with the duration of IAV infection, promoting autophagosome formation in the early stages of infection and inhibiting autophagosome maturation in the later stages (Gannagé et al., 2009). Many studies have demonstrated that autophagy promotes viral self-replication (Zhang et al., 2021; Wang et al., 2020; Wang et al., 2019b). Interestingly, the virus exhibited complex regulatory interactions with autophagy (**Table 1**), as seen with H3N2 and H1N1 viruses (Choi et al., 2019; Gannagé et al., 2009), which could both inhibit and promote autophagy. However, H9N2 and H5N1 viruses typically induce autophagy (Zhang et al., 2021; Zhou et al., 2009; Zhang et al., 2019; Ma et al., 2011; Pan et al., 2014).

**Table 1:** Relationship between IAV and autophagy.

Virus	Autophagy genes	Experimental cells or animals	Impact on Autophagy
A/duck/Hubei/Hangmei01/2006 (H5N1) (HM/06)	LC3	A549	The binding of NP to LC3 promoted vRNP export, while M2 interaction with LC3 boosted infectious viral particle production (Wang et al., 2019b).
A/Hong Kong/8/68 (H3N2) (A/HK/68)	Atg5, Atg7, Beclin 1, Ulk1, Rb1cc1, Maplc3a, Maplc3b, Gabarap, Atg14, Uvrag	Alox5^-/-^B/Atg7^-/-^mice	Autophagy played a key role in maintaining the survival of memory B cells and regulating their protective antibody response to viral infection (Chen et al., 2014).
A/WSN/1933 (H1N1)	LC3	A549	During early-stage IAV induced autophagic degradation of antioxidant enzyme SOD1, leading to increased reactive oxygen species (ROS) production and enhanced viral infectivity (Jung et al., 2018).
A/WSN/33 (H1N1), A/chicken/Beijing/04 (H9N2)	LC3-I/LC3-II, Beclin 1	MDCK	The virus increased the amount of LC3-II and enhanced the autophagy flux (Zhou et al., 2009).
A/Aichi/68 (H3N2), A/WSN/33 (H1N1), A/PR8/34 (H1N1)	Atg8/LC3-II	A549, MDAMC, HaCat, MLE-12, MDCK, HeLa	IAV inhibited the fusion of autophagosome and lysosome through its M2 protein, thus inhibiting the autophagy pathway and promoting cell apoptosis (Gannagé et al., 2009).
H5N1	p-Akt, p-S6, S6, LC3-II	Tsc2^-/-^ and Pten^-/-^ MEF	The H5N1 virus caused autophagy cell death by inhibiting the mTOR signaling pathway (Ma et al., 2011).
A/Hongkong/8/68 (H3N2), A/Wisconsin/33 (H1N1)	LC3, p62	HEK293, TREx-293, MCF-7, MDCK	The M2 protein in the virus could block the fusion of autophagosomes with lysosomes (Ren et al., 2016).
A/WSN/33 (H1N1), A/PR8/34 (H1N1)	LC3	Atg7^-/-^ MEFs, A549, MDCK, HeLa	Autophagy participated in the accumulation of viral components (RNA and protein) of IAV, which was linked to Hsp90 and mTOR signaling pathways (Liu et al., 2016).
H3N2	p-mTOR, LC3, ATG5, p62	C57BL/6 male mice, A549	The combination of LPS with viruses activated autophagy (Wang et al., 2023).
A/Virginia/ATCC2/2009 (H1N1), A/Weiss/43 (H1N1), A/California/2/2014 (H3N2)	P62	A549, MDCK, SPF BALB/c mice	The virus reduced the autophagic protein p62 (Ge et al., 2023).
A/duck/Hubei/Hangmei01/2006 (H5N1) (H5N1/HM) (PB1-F2_HM_), A/PR8/H1N1 (PB1-F2_PR8_)	LC3	HEK 293T, A549, MDCK	PB1-F2 interacted with LC3B to induce mitochondrial autophagy (Wang et al., 2021).
H9N2	LC3, Atg5	A549	H9N2 induced autophagy, and autophagy regulated oxidative stress via the Akt/TSC2/mTOR signaling pathway, thereby promoting the replication of the virus (Zhang et al., 2021).
A/new.Coledonia/20/1999 (H1N1), A/Jilin/9/2004 (H5N1)	mTOR, p-mTOR, Akt, p-Akt, S6, p-S6, LC3, TSC2, Atg5, Atg6	A549	The HA protein of the H5N1 virus induced autophagy, and inhibiting autophagy ameliorated virus-induced acute lung injury (Sun et al., 2012).
A/PR/8/34 (H1N1), A/FPV/Rostock/34 (H7N1)	LC3-I/LC3-II, Beclin 1	MDCK, CV-1	NS1 indirectly stimulated autophagy by up-regulating the synthesis of HA and M2 (Zhirnov and Klenk, 2013).
A/PR/8/34 (H1N1), KBPV-VR-32 (H3N2)	autophagosome	MDCK	Virus induced autophagy in MDCK cells (Choi et al., 2019).
A/Vic/3/75 (H3N2, VR-822), A/PR/8/34 (H1N1, VR-1469)	ATG-5, ATG-7 and LC3	MDCK	The expression level of the autophagy gene (ATG-5, ATG-7 and LC3) increased significantly after the virus infection (Gansukh et al., 2016).
A/swine/HeBei/012/2008 virus (H9N2 virus)	LC3, p62, Atg5, mTOR, p-mTOR, Akt, p-Akt	A549, BALB/c mice	H9N2 infection resulted in a significant increase in the level of autophagy, and autophagy contributes to the process of viral replication (Zhang et al., 2019).
A/Quail/Hong Kong/G1/97 (H9N2/G1), A/Hong Kong/54/98 (H1N1)	LC3B, Atg5, p62, p70S6K	Human blood macrophages	H9N2/G1 viruses infected cells more effectively induced autophagy than H1N1 viruses (Law et al., 2010).
H5N1	beclin1, Atg5	HEK293T, A549	NF-κB activation enhanced H5N1-induced autophagosome formation, thereby exacerbating H5N1-induced lung inflammation (Pan et al., 2014).

A549, human lung epithelial cells; MDCK, Madin-Darby canine kidney; MLE-12, mouse lung epithelial cells; MDAMC, human breast carcinoma cells; HaCat, human keratinocyte cells; MEF, mouse embryonic fibroblasts; Hela, human cervical carcinoma cell; HEK293, Human Embryonic Kidney 293; MCF-7, Michigan Cancer Foundation-7; TREx-293, Modified HEK293 cell line with T-REx™ (Tight-Regulated Expression) system; CV-1, Monkey Kidney Cells 1.

### 3.1 IAV infection promotes autophagosome formation

Autophagosomes are abundant in cells infected with IAV (Cui et al., 2020). H1N1 virus-induced autophagy in dendritic cells through endocytosis, and autophagy-deficient bone marrow-derived dendritic cells (BMDC) showed significant deficiencies in eliciting innate and adaptive immune responses to H1N1 (Zang et al., 2016). Zhou et al. found that after infection of MDCK cells with either H1N1 or H9N2, fluorescence and electron microscopy observed a significant increase in the amount of LC3-II, which further confirms that viral infection promotes increased autophagosome formation (Zhou et al., 2009). In addition, the H5N1 virus also stimulated the mTOR-related autophagy pathway by regulating TSC2 expression (Ma et al., 2011). Dai et al. found that IAV infection increased the expression of ATG5, ATG12, Beclin1 and ATG9, increased LC3-I to LC3-II conversion, and promoted autophagosome formation (Dai et al., 2012a).

### 3.2 IAV infection inhibits autophagosome

**Figure 3:**
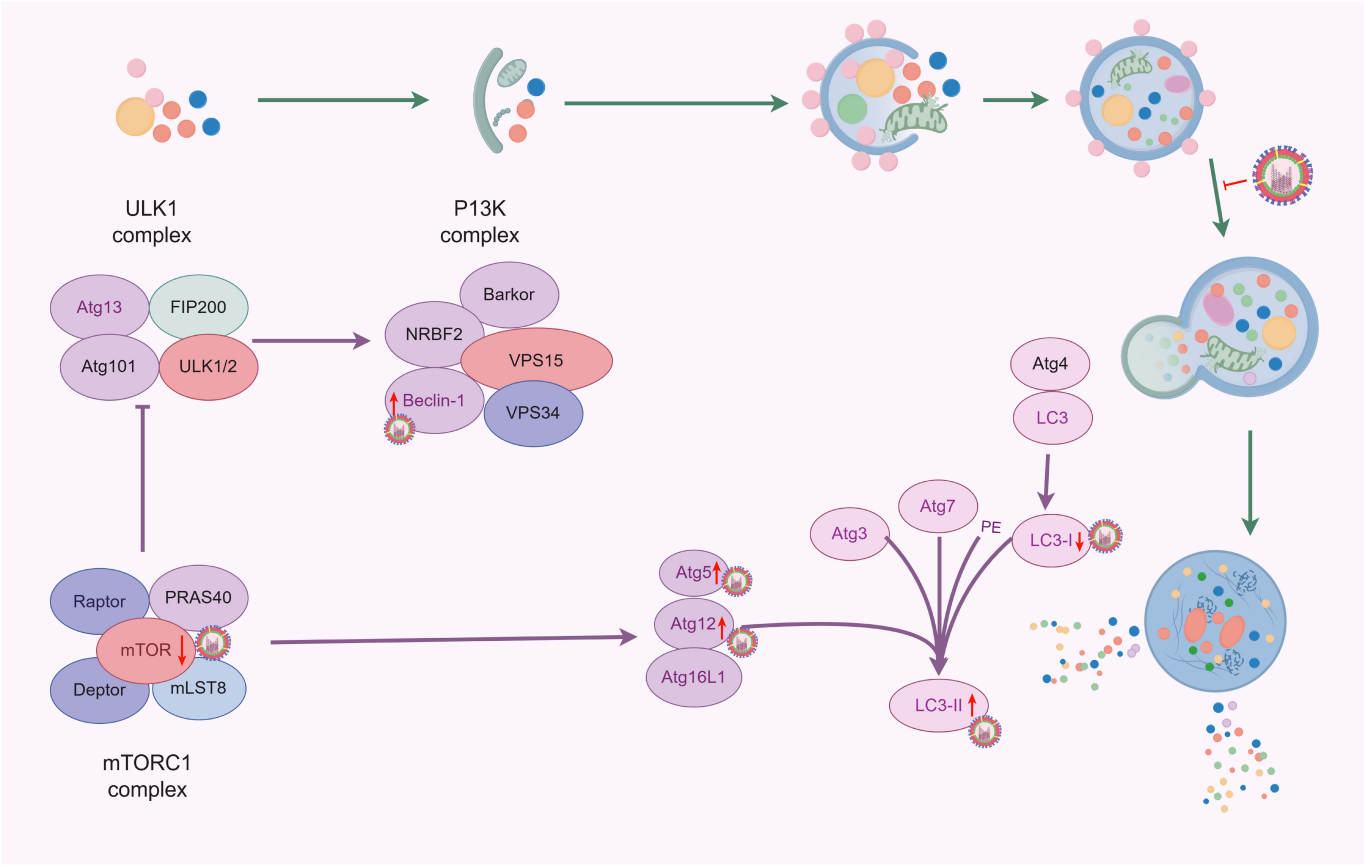
Effects of IAV infection on autophagy (By Figdraw).

Viruses can suppress autophagy by disrupting its initiation signals and altering the formation of autophagosomes, thus evading host immune responses and delaying their clearance. The study found that H1N1 infection in A549 cells significantly reduces the expression of p62 protein within 24 h (Ge et al., 2023). Autophagosomes are typically referred to as transient vesicles that are quickly degraded by lysosomes. Therefore, the accumulation of autophagosomes induced by IAV may indicate an increase in their formation or a decrease in their degradation (Klionsky et al., 2012). The study by Team Gannagé et al. identified M2, the first viral protein that blocks autophagosome degradation, this protein inhibited the fusion process of autophagosomes with lysosomes, thus contributing to the accumulation of autophagosomes in IAV-infected A549 cells (Gannagé et al., 2009). Corresponding studies showed that autophagosomes also accumulated in dendritic cells (DCs) during IAV infection, while the fusion of autophagosomes with lysosomes was inhibited (Deng et al., 2021). Further studies using defective virus-infected cells with knockdown of the M2 protein and detected by the mCherry-GFP reporter system found that the fluorescence intensities of the two groups did not show a significant difference when compared with normal virus-infected DCs, suggesting that the M2 protein may not play a role in blocking the fusion of autophagosomes with lysosomes during IAV-induced autophagy. This is contradictory to the results of previous studies (Deng et al., 2021; Gannagé et al., 2009), probably because of the different experimental methods - the former involves transfection of M2 protein into A549 cells, while the latter involves the use of M2 protein-deficient virus-infected cells. Thus, further studies are needed to elucidate whether the M2 protein is specifically involved in or interferes with the fusion mechanism of autophagosomes and lysosomes. However, IAV infection certainly blocks the fusion of autophagosomes with lysosomes, thereby inhibiting the autophagic process (**Figure 3**).

### 3.3 Effects of PB2, M2, HA and NS1 proteins of IAV on autophagy

PB2, M2, HA, and NS1 proteins are involved in regulating the autophagy process in host cells, and the M2 protein alone can induce the initial formation of autophagosomes (Kuo et al., 2017; Zhirnov and Klenk, 2013; Guo et al., 2023). Gannagé et al. demonstrated that the expression of the autophagosome marker LC3-II was increased in M2-transfected cells, but the fusion of autophagosomes with lysosomes was hindered. Meanwhile, autophagosomes accumulated to a significantly lower extent in virus-infected cells lacking M2. These results suggested that the M2 protein regulated the autophagy mechanism in IAV replication by interfering with the fusion process between autophagosomes and lysosomes (Gannagé et al., 2009). In another study, Liu et al. found that the PB2 protein of IAV interacted with the p62 autophagy receptor, which promoted the viral replication process (Liu et al., 2021). Specifically, depletion of p62 prevented the formation of vRNP aggregates in cells infected with the avian PB2(627E) virus, suggesting that p62 was essential for the formation of vRNP aggregates in virus-infected cells. The avian PB2(627E) virus-induced higher levels of autophagic responses in infected cells compared to the mammalian-type PB2(627K) virus (Liu et al., 2021). Additionally, the HA protein, the major surface glycoprotein of the virus, activated autophagy, with the lysis products of H5 and H7 leading to a significant up-regulation of the LC3-II protein (Zhirnov and Klenk, 2013). Another study showed that heat shock protein 90AA1 (HSP90AA1) on the cell surface was directly bound to the HA1 subunit of IAV and induced autophagy via the AKT-mTOR pathway (Wang et al., 2020). Furthermore, NS1 inhibited apoptosis in the early stages of infection by inhibiting viral replication, and may indirectly stimulate autophagy by increasing the synthesis of HA and M2 proteins (Baskin et al., 2009). These different mechanisms show that viruses have diverse strategies in regulating host cell autophagy, which helps them to adapt to and exploit the host cell environment, thereby facilitating viral replication and survival.

### 3.4 Autophagy promotes IAV replication

**Figure 4:**
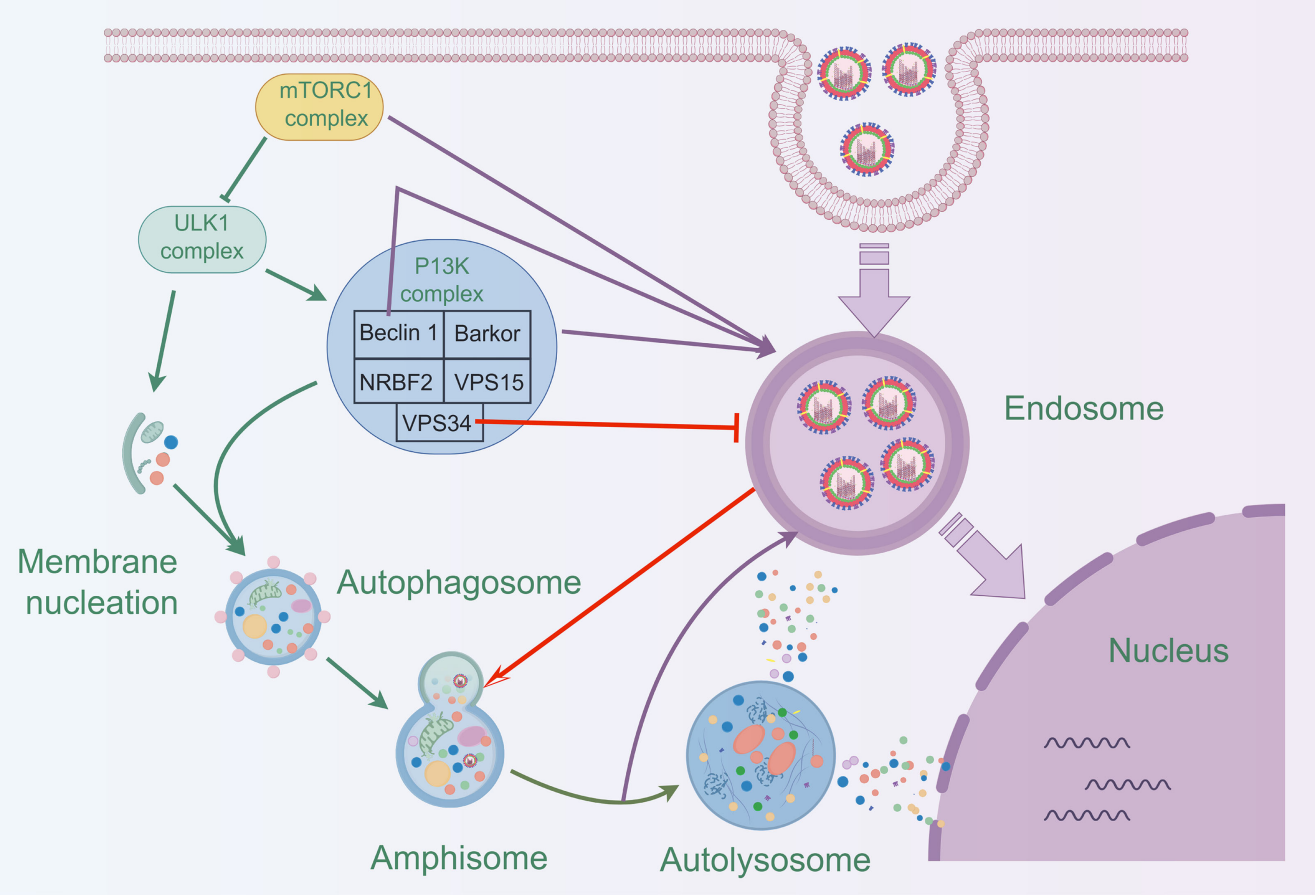
Autophagy mediated IAV replication (By Figdraw).

Autophagy is a cellular protective mechanism that helps cells cope with oxidative stress or inflammatory damage (Ornatowski et al., 2020; Lin et al., 2019), and can also be utilized by viruses to promote their survival and reproduction (**Figure 4**). It has been reported that when H1N1 or H9N2 infected MDCK cells, the level of autophagy was significantly increased, and the virus titers were significantly decreased with pharmacological inhibition (3-methylademine and wortmannin) or RNA interference targeting autophagy, indicating that autophagy actively participated in the replication of IAV (Zhou et al., 2009). Consistent with this, following transfection with the Beclin-1 expression plasmid, the cells showed significantly increased viral titers with the H1N1 virus at 24 h. However, after 48 h, there was no significant difference in viral titers compared to the control group (Feizi et al., 2017). This suggests that increasing autophagy before infection may promote early viral replication. Autophagy influences viral replication not only through the PI3K complex, but also by regulating mTOR and the fusion of autophagosomes with lysosomes (Li et al., 2022; Wang et al., 2023).

### 3.5 Autophagy inhibits IAV replication

Most studies indicate that autophagy promotes influenza A virus (IAV) replication. However, some studies have suggested the opposite, proposing that autophagy inhibits viral replication. One of these studies observed a significant increase in H1N1 titers in VPS34-deficient cells. In contrast, cells that re-expressed VPS34 showed a significant decrease in H1N1 titers (Yu et al., 2019). VPS34, a class III phosphatidylinositol 3-kinase, is crucial for autophagy, particularly in the formation of autophagic vesicles. These findings suggested that restoring autophagy function can effectively reduce the replication of the H1N1 virus within cells. On the other hand, Guo et al. proposed that the fusion of autophagosomes and lysosomes facilitates the degradation of IAV enclosed in autophagosomes, thereby inhibiting viral replication (Guo et al., 2024).

## 4 Antiviral effects of natural products

### 4.1 Safety of natural products

In recent years, natural products have garnered widespread attention as antiviral agents. However, their safety is equally important in both research and clinical applications. Various natural products (such as flavonoids, polyphenols, and polysaccharides) have been evaluated to explore their safety and potential uses in treating viral infections. Trehalose (α-D-glucopyranosyl-α-D-glucopyranoside) is a natural disaccharide approved as a safe food ingredient by the U.S. Food and Drug Administration (FDA) (Schiraldi et al., 2002). Trehalose is widely found in plants, insects, microorganisms, and invertebrates and is recognized as an effective autophagy inducer (Wu et al., 2015). Studies indicated that trehalose promoted the fusion of autophagosomes with lysosomes, preventing viruses from entering exosomes and thus inducing Zika virus degradation (Yuan et al., 2017). Additionally, it increased the expression levels of LC3B-II, Beclin-1, and ATG5/ATG12, thereby inhibiting human cytomegalovirus infection (Belzile et al., 2016). Resveratrol (3,5,4′-trihydroxy-trans-stilbene) is a polyphenol that demonstrates good safety. No significant cytotoxicity was observed in MDCK cells exposed to 10-20 μg/mL of resveratrol. Continuous intraperitoneal injection of resveratrol (1 mg/kg/day) for 7 days in mice also showed no toxicity and effectively inhibited H1N1 virus replication both *in vitro* and *in vivo* (Palamara et al., 2005). Moreover, resveratrol showed an IC_50_ of 24.7 μM against H1N1 and H3N2 viruses in A549 cells, indicating significant antiviral effects (Lin et al., 2015). Additionally, research showed that quercetin 3-rhamnoside (10 μg/mL) did not exhibit noticeable toxicity toward MDCK cells and could effectively inhibit IAV infection (Choi et al., 2009). Although these natural products exhibit good safety profiles and significant antiviral activity at recommended concentrations, their practical application requires careful consideration. It is particularly important to monitor their long-term safety and effectiveness against different types of viruses and stages of infection. A thorough evaluation is crucial for guiding further research and clinical application of these natural products.

### 4.2 Effective anti-IAV components in natural products

**Figure 5:**
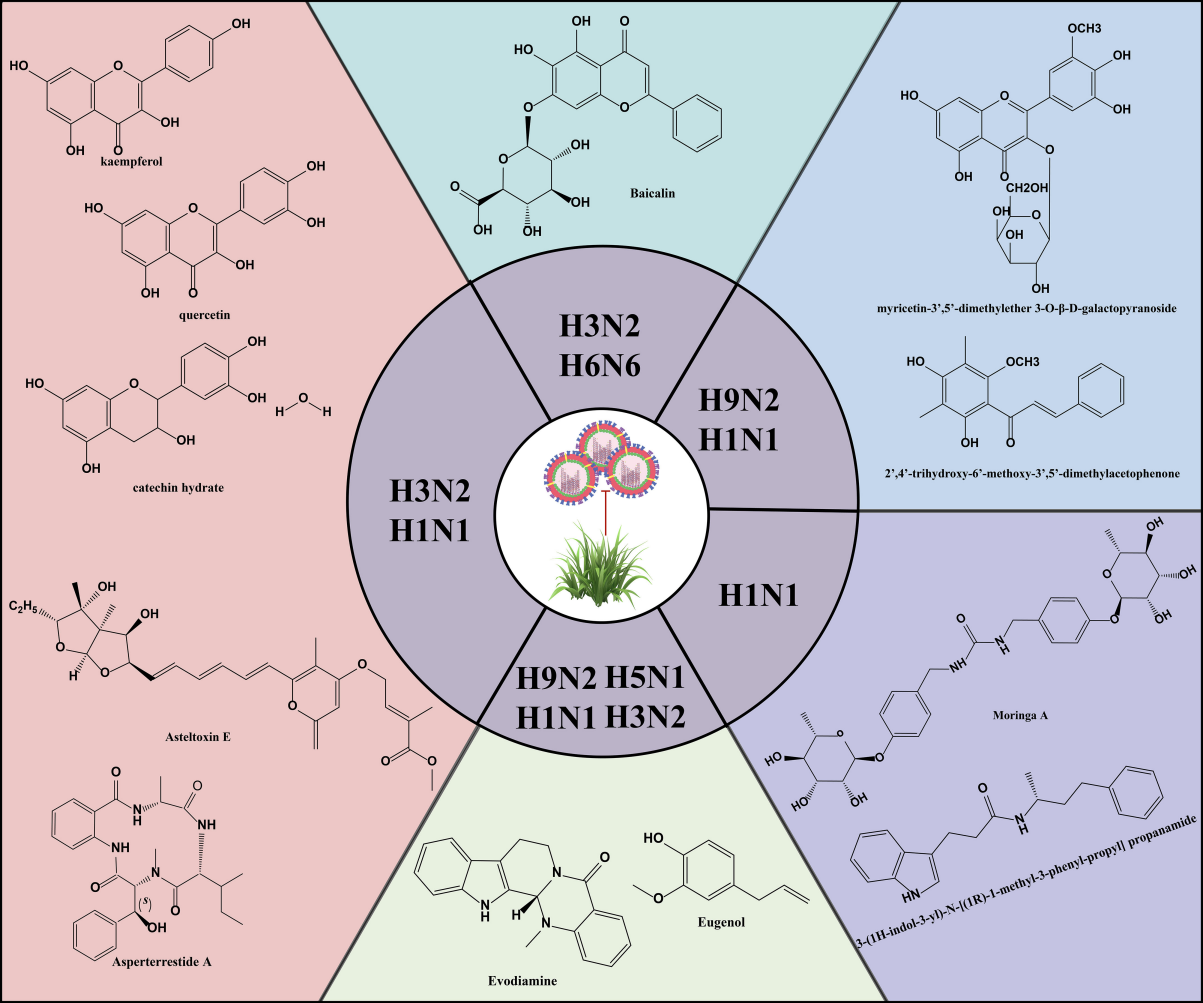
Structures of representative natural products with anti-IAV activity.

Natural products are derived from the constituents of animals, plants, microorganisms, insects and marine organisms or their metabolites, and are known for their wide range of sources, diverse compositions, unique structures, low side effects and low susceptibility to drug resistance. In recent years, studies have continued to reveal the activity of natural products such as mycobacterial metabolites, polyphenols, flavonoids, alkaloids and terpenoids in antiviral infections (**Figure 5**) (Li et al., 2024; Li et al., 2024; Zhang et al., 2024). Asperterrestide A inhibited A/WSN/33 (H1N1) and A/Hong Kong/8/68 (H3N2) viruses with half inhibitory concentration (IC_50_) values of 15 μM and 8.1 μM, respectively (He et al., 2013). In addition, a new aspergillus toxin, Asteltoxin E, from *Aspergillus* sp. SCSIO XWS02F40, showed similarly significant inhibitory activity against the H1N1 and H3N2 viruses, with an IC_50_ of 3.5 ± 1.3 μM and 6.2 ± 0.08 μM, respectively (Tian et al., 2015). Perovic et al. utilized the ZINC Natural Product database, the ligand-based virtual screening and molecular docking technique, 3-(1H-indol-3-yl)-N-[(1R)-1-methyl-3-phenyl-propyl] propanamide was successfully identified as a promising anti-influenza virus natural drug candidate (Perovic et al., 2024). *Aloe vera* ethanol extract showed the potential to inhibit autophagy in H1N1 or H3N2 infected MDCK cells (Choi et al., 2019). The main components were found to include quercetin, catechin hydrate and kaempferol by ultra-performance liquid chromatography-tandem mass spectrometry (UPLC-MS/MS). Molecular docking simulations showed that these components are bound with high affinity to M2 proteins. M2 proteins can inhibit the fusion of lysosomes and autophagosomes, thus further inhibiting autophagy (Deng et al., 2021). Therefore, Choi et al. hypothesized that the inhibition of viral infections by *Aloe vera* ethanol extract may be through affecting M2 proteins in the regulation of autophagy-related mechanisms (Choi et al., 2019). However, further in-depth studies are needed to verify this putative mechanism. The ethanolic extract of *Cleistocalyx operculatus* leaves contains compounds such as 2’,4’-trihydroxy-6’-methoxy-3’,5’-dimethylacetophenone and myricetin-3’,5’-dimethylether 3-O-β-D-galactopyranoside, which have demonstrated inhibitory activity against IAV including H1N1 A/PR/8/34, H9N2 A/Chicken/Korea/O1310/2001, wild-type novel swine influenza, and oseltamivir-resistant strains (H274Y mutation) (Ha et al., 2016). These findings not only enrich the candidate pool of anti-IAV drugs but also provide new directions and possibilities for the development of novel antiviral drugs and therapeutic strategies.

### 4.3 Autophagy regulation by natural products

#### 4.3.1 Regulation of autophagy-related signaling pathways by natural products

Studies in recent years have gradually revealed the potential role of natural products in regulating cellular autophagy signaling pathways (Chen et al., 2024; Chen et al., 2024; Jung et al., 2024). Salidroside was found to inhibit the autophagy process by binding to key proteins such as mTOR, SIRT1 and AKT1 with high affinity through the systemic pharmacological study of Chinese medicine by Chai et al (Chai et al., 2024). Baicalin can affect the expression of LC3-II proteins and thus trigger autophagy by regulating signaling pathways such as PI3K/Akt/mTOR, ERK1/2 and β-catenin (Abbott and Ustoyev, 2019). Selenizing *Astragalus* polysaccharide (Liu et al., 2018) and *Platycodon grandiflorus* polysaccharides (Xing et al., 2021) regulate autophagy triggered by viral infection by modulating the Akt/mTOR signaling pathway. The total flavonoids of *Abelmoschus manihot* were able to regulate autophagy through the AMPK/mTOR pathway (Zhang et al., 2024). And other natural products such as *Atractylodes macrocephala* polysaccharide (Qi et al., 2024), total flavone of Abelmoschus Manihot (Zhang et al., 2024), luteolin, apigenin, and chrysin (Lo et al., 2024), Hesperidin (Kumar et al., 2023), phosphorylated *Codonopsis pilosula* polysaccharide (Ming et al., 2020) and *Chrysanthemum indicum* polysaccharide (Ming et al., 2019) also showed the ability to regulate autophagy. These findings suggested that natural products could regulate autophagy by affecting cellular signal transduction pathways such as AMPK/mTOR and PI3K/Akt/mTOR, which was important for the development of novel anti-IAV therapeutic strategies. However, whether they possess antiviral activity remains to be clarified through further research to elucidate the complex relationship between these factors. Therefore, we will explore natural products that exhibit both autophagy-regulating and anti-IAV properties.

#### 4.3.2 Natural products targeting autophagy to prevent IAV infection

With the increase of global public health awareness and the emergence of viral mutations, the research of natural products in the field of antiviral will be of even greater significance. Autophagy is almost a process that promotes viral replication by observing the relationship between autophagy and the IAV in sections 3.4 and 3.5, and therefore inhibiting autophagy may help to suppress viral infection. This finding provides a theoretical strategy for the development of antiviral drugs.

##### 4.3.2.1 Flavonoids

Natural products that can regulate autophagic processes have become a hot topic in research on the treatment of viral infections, especially flavonoids (Chang et al., 2020; Song and Choi, 2011). Baicalin inhibited H3N2 or H6N6 virus-induced autophagy and down-regulated the expression levels of autophagy-related factors (LC3II, ATG12 and ATG5) in RAW264.7 or A549 cells, suggesting that baicalin may reduce viral infectivity by inhibiting autophagy (Zhu et al., 2015; Yang et al., 2024). In addition, procyanidin, a natural product enriched in a variety of traditional Chinese medicines, has been shown to significantly inhibit the accumulation of LC3II and the aggregation of GFP-LC3, as well as reduce the expression levels of Atg7, Atg5 and Atg12 in MDCK cells. Through further molecular mechanism studies, procyanidin inhibited the formation of Atg5-Atg12/Atg16 heterotrimer and the dissociation of beclin1/bcl2 heterodimer, which provided a scientific basis for their use as novel anti-IAV drugs (Dai et al., 2012a). These *in vitro* experiments above provided a preliminary understanding of the role of natural products at the cellular level, but did not fully reflect their actual effects in complex organisms. *In vitro* experiments usually lack factors such as biotransformation, metabolism and drug transport *in vivo*. Therefore, the results of *in vitro* environments need to be further validated for their actual effects and safety through animal models and clinical trials.

The activation of toll-like receptor (TLR) 4 plays an important role in IAV infection (Ma et al., 2024; Wang et al., 2022). He et al. constructed a human TLR4 promoter dual luciferase reporter gene assay system and screened 161 TCM-based TLR4 inhibitors. The results showed that could effectively inhibit viral infection and replication. Further studies showed that Apigetrin not only effectively reduced the accumulation of autophagy markers LC3-II and p62 *in vitro*, but also showed similar effects *in vivo*, which reinforces its reliability as an antiviral therapeutic candidate (He et al., 2023). Meanwhile, other studies have found that overexpression of TLR4 promotes autophagic activity (Wang et al., 2020). Unfortunately, no study has yet definitively confirmed whether the natural product regulates autophagy by inhibiting TLR4, thereby inhibiting IAV infection and transmission. Nevertheless, these findings provide a theoretical basis for the possibility that natural products may regulate autophagy by inhibiting TLR4, thereby inhibiting IAV infection and transmission, and opening up new research directions for exploring alternative drug targets for autophagy regulation.

##### 4.3.2.2 Other natural products

In addition to the above flavonoids, natural products such as Tanreqing injection (Guo et al., 2024), Moringa A from Moringa oleifera seeds (Xiong et al., 2021), gallic acid (Chang et al., 2024b), *Isatis indigotica* (Chang et al., 2024a), eugenol (Dai et al., 2013b) and Hochuekkito (Takanashi et al., 2017) have been recognized for their ability to anti-IAV virus infections by modulating autophagy. The reduced production of H1N1 virus proteins (M1, M2, and NP proteins), the significant decrease in LC3B II conversion, and the decrease in the accumulation of autophagosomes with the increase in gallic acid concentration in gallic acid-treated H1N1 IAV suggest that gallic acid maybe suppresses H1N1 viral infectivity by inhibiting the autophagy pathway and suppressing the production of virulent M1, M2, and NP proteins (Chang et al., 2024b).

Interestingly, Dai et al. developed a new drug screening method based on autophagy signaling pathway to screen novel anti-IAV drugs using a bimolecular fluorescence complementation-fluorescence resonance energy transfer (BiFC-FRET) assay. This approach not only enables the screening of potential anti-IAV drugs but also elucidates the molecular mechanisms underlying their action, thereby providing a crucial foundation for future drug development. Evodiamine (the main active ingredient of Evodia rutaecarpa Benth) (Dai et al., 2012b), 23-(S)-2-amino-3-phenyl-propanoyl-silybin (an amino acid derivative of silymarin) (Dai et al., 2013a), and eugenol (the main active ingredient of *Syzygium aromaticum L.*) (Dai et al., 2013b) were found to have anti-IAV potential, each through distinct mechanisms. Among them, Evodiamine and 23-(S)-2-amino-3-phenyl-propanoyl-silybin inhibit IAV replication by inhibiting the formation of the Atg5-Atg12/Atg16 complex, whereas eugenol exerts its anti-IAV effect by effectively blocking the dissociation of the Beclin1-Bcl2 complex. Beclin1 is a key regulator of autophagy, while Bcl2 is an important inhibitor of Beclin1 (Dai et al., 2013b). Normally, the binding of Bcl2 to Beclin1 inhibits the initiation of autophagy. Therefore, Dai et al. concluded that eugenol inhibited autophagy by preventing the dissociation of this binding, thus exerting its anti-IAV ability.

Differently, Moringa A from Moringa oleifera seeds inhibited the expression and nuclear translocation of transcription factor EB (TFEB) (Xu et al., 2020), a key regulator of autophagy and lysosomal biogenesis. This suggested that Moringa A impaired autophagy by blocking lysosome production (Xiong et al., 2021). These studies not only reveal the potential application of natural products in the treatment of viral infections but also provide important clues for understanding the mechanism of autophagy in viral infections. The above findings indicated that natural products could regulate autophagy through various mechanisms to inhibit viral replication, providing important clues to our in-depth understanding of the autophagy mechanism in viral infections.

Although current research showed that certain natural products could modulate autophagy and exhibited potential against IAV, it remained to be determined whether their antiviral activity was directly related to their effects on autophagy. In this review, we observed that the focus of research on these natural products has primarily been on their ability to inhibit IAV by suppressing autophagy, which is an intriguing and noteworthy finding. However, natural products may exhibit both autophagy-promoting and autophagy-inhibiting effects to achieve antiviral or virus-suppressing outcomes. Therefore, future research should comprehensively investigate the relationship between the antiviral mechanisms of natural products and their modulation of autophagy. This will help uncover the complex mechanisms involved and provide a solid scientific basis for developing effective antiviral drugs.

## 5 Conclusion and prospects

Natural products provide new strategies and directions for the development of novel antiviral drugs by regulating the function of autophagy in viral infections. However, it is noteworthy that natural products like polysaccharides (Liu et al., 2018; Ming et al., 2020, 2019), saponins (Li et al., 2019; Cheng et al., 2006), polyphenolic compounds (Wang et al., 2023; Zhu et al., 2023), and alkaloid (Liu et al., 2020; Wang et al., 2018) have demonstrated the ability to modulate autophagy and exhibit antiviral activity against other viruses, while researchers have not extensively explored their connection with autophagy and IAV. Therefore, future studies should investigate the role of these natural products in regulating autophagy triggered by IAV. Additionally, advanced technologies should be developed, such as BiFC-FRET and histological techniques, which will facilitate more efficient and rapid screening of natural products for their antiviral and autophagy-regulating properties.

Natural products exhibit potential for bidirectional regulation of autophagy. On one hand, they can inhibit autophagy to prevent IAV replication; on the other hand, some natural products can promote the fusion of autophagosomes with lysosomes, accelerating the degradation of viruses within autophagic lysosomes. This bidirectional mechanism allows natural products to modulate autophagy levels to restore homeostasis. However, accurately quantifying autophagy levels is crucial due to the dynamic nature of viral infections and autophagy. The different influenza strains, infection duration, and individual responses to autophagy necessitate the use of standardized techniques such as immunofluorescence staining, electron microscopy, western blotting, transcriptomics, and proteomics to measure autophagy markers. Additionally, gene editing technologies or pharmacological interventions should be used to regulate the expression of autophagy factors, and suitable animal models and *in vitro* cell models should be established to simulate different states of autophagy. These approaches will help to explore the antiviral mechanisms in greater depth.

Autophagy’s effects on viruses are bidirectional and involve complex interactions with oxidative stress, apoptosis, and inflammatory responses (Ishfaq et al., 2021; Hsueh et al., 2024). However, it is unclear how IAV proteins coordinately regulate autophagy to support their propagation and how the host can IAV replication by modulating autophagy. To gain a deeper understanding of the mechanism of autophagy in viral infections, future studies need to pay more attention to the specific roles of autophagy in different stages of infection, especially how the host influences viral replication and propagation by regulating autophagy. This will enable a more comprehensive understanding of the role of autophagy and provide stronger support for future drug development.

**Figure 6:**
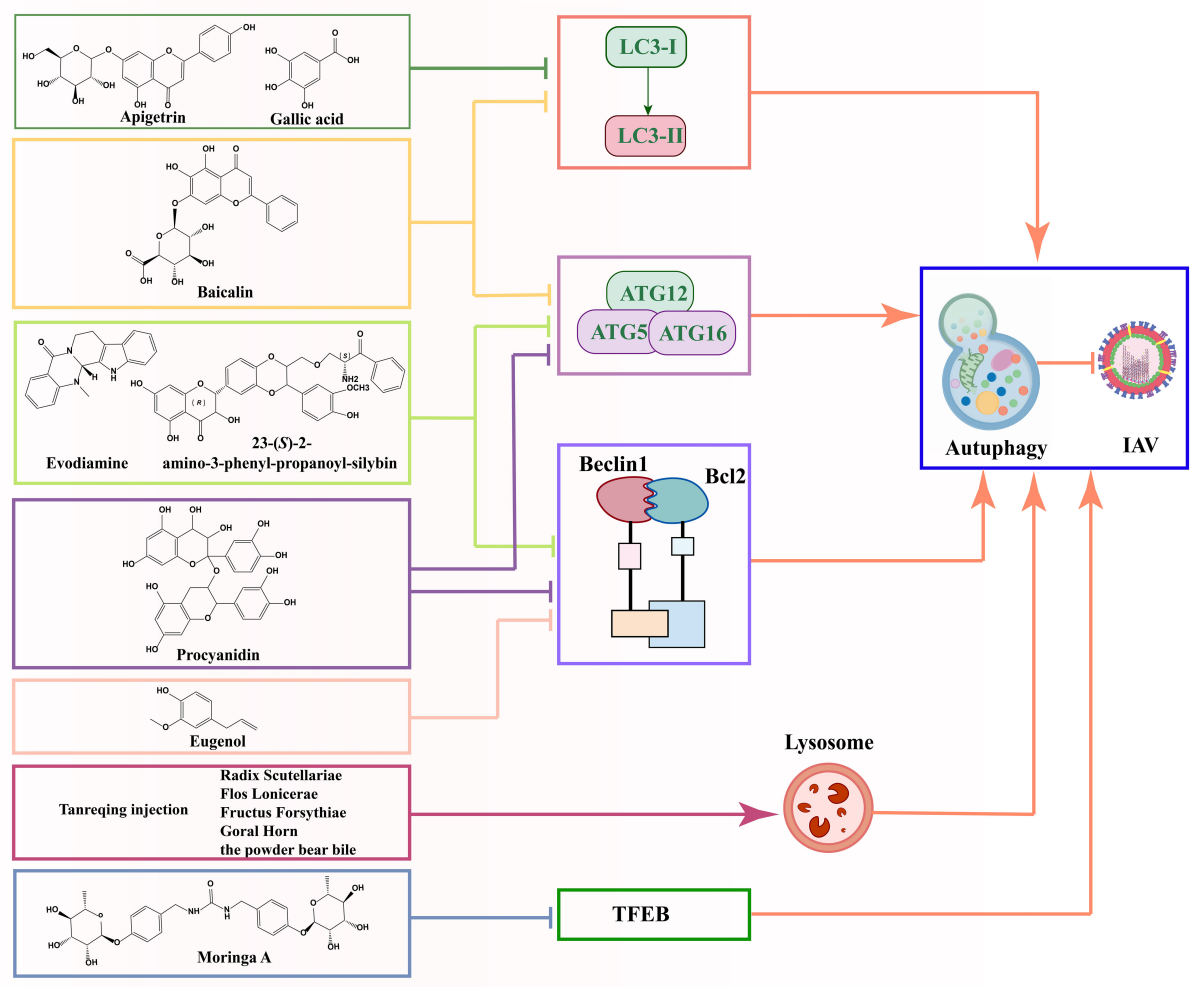
Schematic diagram of the mechanism by which natural products prevent IAV infection via autophagy pathway.

Most of the current research on natural products and autophagy regulation focuses on single targets, such as LC3II, Atg5-Atg12/Atg16 heterotrimer, beclin1/bcl2 heterodimer, and research is still in its infancy (**Figure 6**). There is a need for more comprehensive research on multiple targets, phenotypes, and pathways to better reveal the deeper roles of natural products. Despite the advantages of natural products, such as multi-targeting and low toxicity, they face challenges like low bioavailability, poor absorption, and rapid metabolism, which can impact drug efficacy. Additionally, the unclear composition and structure of herbal extracts (e.g., flavonoids and polysaccharides) limit research progress. Addressing these challenges requires strengthening the research on the composition and structure of natural products. The targeting and efficacy of drugs can be improved through structural modification and the development of related derivatives. In addition, toxicity studies also need improvement, as some natural products may exhibit toxicity at high doses.

The modulation of autophagy by natural products can affect viral infections through various mechanisms, including endocytosis, autophagosome formation, and maturation. Therefore, selecting natural products as therapeutic agents requires consideration of appropriate delivery strategies. These strategies should be based on the developmental stage of autophagy and its role in different periods of viral infection. In summary, natural products show promise in modulating autophagy and antiviral, further research is essential, particularly on the signaling pathways and mechanisms of autophagy in viral infections, to uncover new therapeutic potentials of plant compounds.
